# The recognition and assessment of pain in people with profound intellectual disabilities by nurses: An appreciative inquiry

**DOI:** 10.1177/17446295241303192

**Published:** 2024-11-22

**Authors:** Maeve Goodall, Kate Irving, Mary Nevin

**Affiliations:** 8818 Dublin City University, Ireland

**Keywords:** Assessment, awareness, pain, profound intellectual disabilities, tool

## Abstract

Individuals with profound intellectual disabilities are non-verbal and reliant on carers for pain recognition, assessment and management. Pain is a multifaceted and interconnected experience. Assessment tools designed specifically for this population are needed. This study aimed to develop methods for improved pain care practices by nurses. This qualitative study followed the four phases of appreciative inquiry; Discovery, Dream, Design, Destiny. Eight nurses were recruited as co-researchers from one Irish intellectual disability organisation. Data were analysed from individual and focus group interviews, using thematic analysis and continuous reflexivity. A pain awareness campaign and RAPPID tool (recognition and assessment of pain in people with profound intellectual disabilities) were developed. Respect for personhood in individuals with profound intellectual disabilities is reflected through holistic approaches to pain assessment. The empowerment of nurses enables positive change. Implicit knowledge can be communicated more proficiently with a formal, collaborative tool.

## Background

People with profound intellectual disabilities make up 2% of the worldwide intellectual disability population (approximately 16 million people) (Maulik et al., 2011), and approximately 4% of the Irish intellectual disability population (Casey et al., 2020). By definition, an individual with profound intellectual disabilities will have extreme difficulties with intellectual and social functioning. They may communicate non-verbally or have very limited verbal ability, and will often need familiar people to assist them with their communication. It is very common for these individuals to have multiple comorbidities, which may include neurological problems, physical or sensory impairments (Bellamy et al., 2010). Within a study by Arvio and Sillanpaa (2003) over ninety nine percent of four hundred and sixty-one individuals with severe to profound intellectual disabilities had multi-morbidities. These may include physical impairments, epilepsy, dysphasia, irritable bowel syndrome and other painful conditions (Gittens and Rose, 2007). Individuals with profound intellectual and multiple disabilities are living longer (Kruithof et al., 2022; McCarron et al., 2017). However, delays in identifying needs and diagnosis of illness is a factor in the remaining inequality of life expectancy between those in the general population and those in the intellectual disability population. People from the intellectual disability population die on average twenty one to twenty four years younger than those in the general population (Blair, 2023). People with intellectual disabilities also have markedly higher rates of painful conditions than the general population, and lower rates of treatment (Baldridge and Andrasik, 2010).

People with profound intellectual disabilities often have idiosyncratic, non-verbal indicators of pain and distress (Chadwick et al., 2019), which may be missed or misinterpreted (Goodall et al., 2023). Even when distress may be recognised, there is a challenge to pinpointing the root cause of this, which can lead to delays in treatment (Adams and Jahoda, 2019). If the presence of physical pain is not recognised in people with profound intellectual disabilities, there is a risk of preventable, advanced illness and death occurring (Donovan, 2002). And so, pain care research for those with profound intellectual disabilities has focused on the physical (Genik et al., 2017; Icht et al., 2021; Petigas and Newman, 2021). There are a plethora of physical pain assessment tools available for people with intellectual disabilities (De Knet et al, 2016; Lotan et. al, 2010; Lotan et al., 2013), including assessments for those who are non-verbal (Solodiuk et. al, 2010; Van der Putten and Vlascamp, 2011). However, there is a need for more holistic and practical pain assessment tools and methods, for this specific population. Pain care is a very complex area for people with profound intellectual disabilities as their individuality and unique communications must be taken into consideration. Evidence suggests that current tools do not allow for fully individualised assessments suitable for this population (Goodall et al., 2023).

Pain is a multifaceted, interconnected and subjective experience; including the psychological, physical, social and emotional (Doody and Bailey, 2017; Ong and Forbes, 2005). Sulmasy (2002: 25) discusses individuals as “beings-in-relationship”, which recognises biological illness as having an affect on all other relational aspects of a person. This concept was coined by Cicely Saunders in the fifties as ‘total pain’ (Saunders, 1993). In order to relieve an individual’s pain, the nurse must consider all experiences and qualities of the person. People with profound intellectual disabilities are highly dependent on carers (Vorhaus, 2014), and so it is the responsibility of these carers to ensure that personhood is bestowed upon and upheld for each individual. Recognising personhood and attending to all aspects of the person’s pain may also improve self-worth for this cohort (Chochinov, 2016).

Challenges to the recognition of pain in individuals within this cohort is compounded by historical beliefs among carers of an insensitivity to pain in people with intellectual disabilities (Symons et. al, 2008; Beacroft and Dodd, 2010). Doody and Bailey (2017) dispute this belief, inferring the possibility of individuals with intellectual disabilities experiencing pain in a more intense way than those in the general population. Current available tools do not address the issue of pain severity for non-verbal individuals with intellectual disabilities (Goodall et al., 2023). There is a need for pain beliefs to be reviewed with staff, to encourage improved pain care (Beacroft and Dodd, 2010).

The aim of this study was to empower nurses, through an appreciative inquiry approach, to creatively develop methods of positive change in pain care practices for individuals with profound intellectual disabilities.

## Methods

### Appreciative inquiry

Appreciative inquiry was developed as a research philosophy and methodology by Cooperider (1986). It adopts a solution focused, strengths-based approach (Cooperider et al., 2008). Appreciative inquiry was originally developed as an approach to large organisational change (Cooperider et al., 2008) and has since become a very adaptable, flexible approach to qualitative research in healthcare (Trajkovski et al., 2013). This methodology is grounded in social constructionism (Yudarwati, 2019), recognising the importance of collaboration and shared language between the co-researchers with experiences of the phenomenon in question (Cooperrider et al., 2008). Throughout an appreciative inquiry study, there is a focus placed on the positive without disregarding the challenges (Reed, 2007).

This collaborative approach and joint engagement, strives to create change through an appreciation of what works well, in order to build on this, creating improvements in the area of interest (Reed, 2007). Within an appreciative inquiry, the participants of the study are considered co-researchers, who have a very active role in the co-creation of knowledge (Reed, 2007). The co-researchers in this study were empowered to lead the co-creation of knowledge and methods of change.

Appreciative inquiry is guided by a 4D cycle; discovery, dream, design and destiny (Cooperrider et al., 2008). The discovery phase aims to uncover ‘the best of what is’ (Reed, 2007: 74). Co-researchers deeply reflect on their own practice (Drayton et al., 2021) and share stories of successes, creating a baseline for improvement (Griggs and Crain-Dorough, 2021). The dream phase aims to create a vision of the very best of what could be. Within the design phase of the study, methods of working towards dreamed visions are developed. These methods are put into practice and evaluated through the destiny phase of appreciative inquiry.

Appreciative inquiry has been found to be effective in creating improvements within healthcare settings (Drayton et al., 2021). With all of the challenges and barriers to best possible pain care for this population, it was beneficial within this study to create a positive stance which appreciated the skills and efforts of the co-researchers. This empowered the co-researchers to become agents for change and allowed for active engagement in the process. Nurses value collaboration (Affleck et al., 2022). Within this study the co-researchers collaborated with each of their respective teams in order to gain as much input and take advantage of a wide range of expertise for the appreciative inquiry process. The co-researching nurses were fully involved in all stages of the appreciative inquiry, with autonomy to lead the change. They each shared their experiences; collected data within their respective teams; envisioned what best practice in pain care looked like and co-designed and developed methods of creating improvements to care.

### Ethics

Ethical approval was received from both the Dublin City University Ethics Committee, reference: DCUREC/2022/199 and the intellectual disability organisation’s ethical review board.

### Recruitment

There is much variability in co-researcher recruitment in appreciative inquiry studies within healthcare, ranging from three co-researchers (Carter et al., 2007) to one hundred and twenty (Seebohm et al., 2010) and above. Appreciative inquiry, as a methodology, is adaptable to the study and its aims (Trajkovski et al., 2013). This study aimed to use a deeply reflective process throughout, gaining an understanding of current practices and values. It required a high level of motivation, and engagement with the co-researchers in the co-creation of knowledge and co-production of materials. For these purposes, a small sample size was required. As previously described in the background, Belamy et al. (2010: 223) conceptualisation of a profound intellectual disability was used for this study.

Invitation to participate in the study was offered by email to all nurses working within one Irish intellectual disability organisation. This recruitment process was assisted by a gatekeeper. All possible candidates were met by MG in person, online or over the phone to review the plain language statement, inclusion/exclusion criteria and answer any questions the potential co-researchers had. Snowball sampling was also used, some candidates for inclusion passed the researchers details to other nurses whom they felt may be suitable or interested in partaking in this study. Written, informed consent was received from each participating nurse.

Eight co-researchers were recruited for the study, from different care areas within the intellectual disability service, residential care (n=6), respite care (n=1) and cross service care (n=1), covering both day and nights. Two of the co-researchers were recruited via the snowball sampling technique. The co-participants included six registered nurses in intellectual disabilities, one registered general nurse and one dual trained registered nurse in intellectual disabilities and mental health. The dual trained nurse from a residential care area engaged with phase one and two of the study before withdrawing due to terminating her employment with the participating organisation. The variety of roles that these co-researchers had included staff nurse, management and specialist nursing. There was an extensive amount of experience within the co-research team, with six of the nurses having over ten years of post-registration experience. Through reflection and discussion with MN and KI, the inclusion/exclusion criteria were defined (see Table 1.).

**Table 1.** Inclusion/Exclusion Criteria.

Inclusion Criteria
The person was a registered nurse of any nursing division in Ireland
The person was currently working with at least one adult with a profound intellectual disability
The person was working in any area of care within the included organisation in any nursing role
Exclusion Criteria
The person was an informal carer, health care worker, social care worker or other health care professional
The person was not currently working with at least one adult with a profound intellectual disability

### Data collection

**Figure 1.**
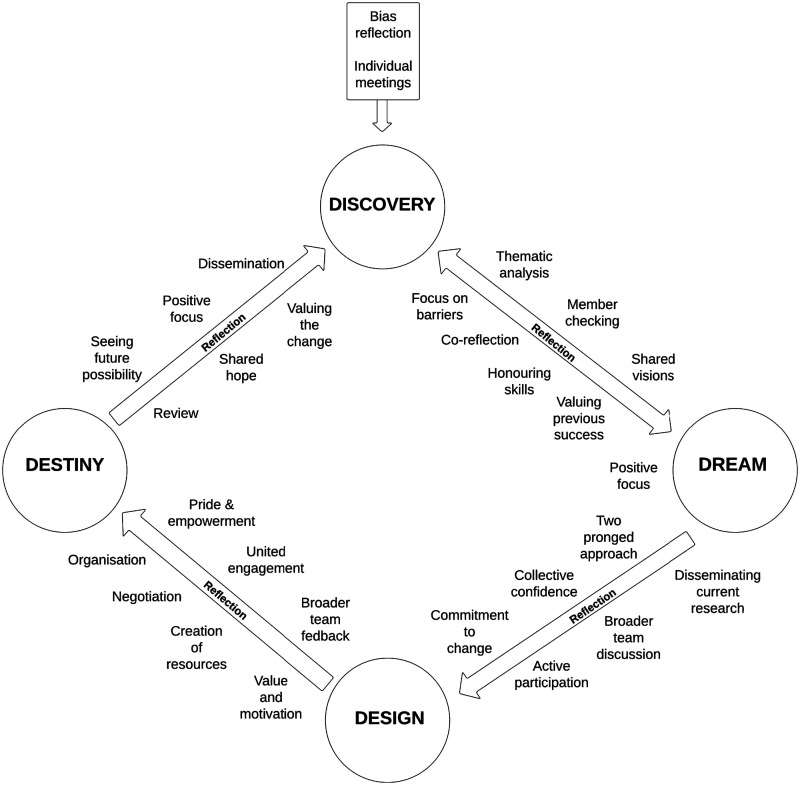
Appreciative Inquiry Process.

Data were collected through semi-structured individual and focus group interviews. These were recorded and transcribed. Each individual interview lasted approximately sixty minutes and each focus group interview lasted approximately ninety minutes. From the discovery and dream phases of the study, the co-researchers chose to develop two strands of dreamed practices. In order to do this, separate interviews were held, focusing on each strand. MG shared information between these two groups of interviews, allowing for feedback and inclusion of all of the co-researchers. Designed documents and resources were also shared with the team of co-researchers via email. Throughout the design phase of the study, the co-researchers involved their staff teams, through discussions at team meetings, in the collection of data. Data were further collected from eleven focus group interviews and six individual interviews, held between March 2023 and March 2024 (see Table 2.); through the four phases of this appreciative inquiry study (see Figure 1). Due to the shift working nature of the co-researcher's roles, it was challenging to coordinate dates and times that suited the full group. Within the first stage of the appreciative inquiry study (discovery), the co-researchers were challenged to reflect on their skills and current practices that are working well in this area of care. Within the second phase of the study (dream), they were asked to imaginatively explore what best practice in pain care for this population would look like to them. Following this, wiithin the third phase of the study (design), the co-researchers were encouraged to actively develop and co-create methods of change. They were again asked to reflect on the study itself and it’s outcomes within the fourth phase (destiny).

**Table 2.** Data Collection - Interviews.

Phase	Data collection - Engagement with co-researchers	Duration period
Discovery/Dream	4 Focus Groups (n=5, n=2, n=3, n=5) Individual interviews: n=1	3 months
Design	5 Focus Groups (n=3, n=5, n=2, n=5. N=2) Individual interviews: n=2	6 months
Destiny	2 Focus Groups (n=2, n=2) Individual interviews: n=3	2 months

### Data analysis

Thematic analysis (Clarke and Braun, 2017) was completed for the discovery and dream phases of the study, using the data software NVivo. Codes were developed from the transcribed interviews and these were grouped into themes. These themes were member checked and discussed in detail with MN and KI. Further analysis was completed by deep reflexivity between the co-researchers and MG within and between interviews. As the co-researchers moved towards the third stage of the appreciative inquiry study (design), creative methods were also used. The researcher and the co-researchers created visual representations of the data collected from stage one and two of the study viamind maps within the interviews. This guided the next phase of the study. Data gathered throughout the study, within their respective services and teams, by the co-researchers were analysed in the focus group interviews. Analysis of all phases of the study was reviewed and discussed with MN and KI.

## Findings

### Discovery/dream

There were four focus group interviews and one individual interview (see Table 2), to uncover the best of current practice in the area of pain care for individuals with profound intellectual disabilities, and to envision the best of what could be. These interviews were positively focused, whilst recognising the challenges and barriers to best care. The six themes that emerged from these interviews were; unconditional positive regard, honouring of relationship, creative best practice, pain through a competing lens, accurate assessment – an impossible task?, and medicating pain – oversimplified and undervalued?

### Unconditional positive regard

The co-researchers explored current practices within their differing care areas. They expressed their respect for and appreciation of each person with profound intellectual disability’s individuality and right to personhood. There was an expression of respect for each individual with profound intellectual disabilities’ ability to communicate, and a recognition that learning to interpret this cohort’s individual forms of communication was the role of the nurse.

“I think it’s amazing to see how they progress through life with all of the barriers that they’re presented” [Co-researcher 3]

### Honouring of relationship

The co-researchers expressed a strong belief of the importance of developing and maintaining a positive and trusting relationship with people in this cohort, in order to recognise their distress. The co-researchers explained that without a formal method of the recognition and assessment of pain, this can become a missed area of care. They also shared stories of the remaining presence of historical beliefs of reduced pain experiences in people with intellectual disabilities, and the challenges of communicating their unique knowledge of each individual both within and outside of their immediate area of care.

“We’ve had somebody who’s non-verbal and they’ve a profound disability, they still communicate. They’ll still make a vocalisation, and this vocalisation means that and this one means that, and it’s not until you know them that you figure it out” [Co-researcher 7]

### Creative best practice

The importance of adapting to the needs and preferences of individuals within this cohort in daily care was emphasised. The co-researchers described methods they have used to create a safe and comfortable atmosphere for the completion of medical interventions in a more successful manner with individuals with profound intellectual disabilities under their care. The co-researchers also described a need for autonomy and the expectation to seek opportunities to upskill due to the changing needs of individuals that are under their care.

“What way can we get around this?” [Co-researcher 4]

### Pain through a competing lens

Although the co-researchers discussed their recognition of pain as a multifaceted, interconnected experience; they stated that there is a priority in the recognition of physical pain, in order to prevent serious medical conditions and death.

“ The first thing….. Is there something medically wrong? And psychology would even say that. “Have we ruled out the medical?”. Rule that out and then - Ok we are clinically fine. Is it emotional? Psychological? Behavioural? …….it’s always medical first.” [Co-researcher 4]

### Accurate assessment – an impossible task?

Assessing the presence of pain was not viewed as an impossible task by the co-researchers, if completed by a regular staff member who has a relationship with the individual being assessed. Without knowledge of the often idiosyncratic and subtle behavioural expressions of pain and distress an individual with profound intellectual disabilities may use, the co-researchers expressed that accurate recognition and/or assessment of pain may not be possible. The co-researchers also expressed that it could be challenging to pinpoint the source of the person’s distress after recognising that it is present, with or without knowledge of the individual.

“We need to be familiar with them, and we need to know that an eye twitch or a grimace or shoulder twitch or whatever their sign is, that that means stop”

[Co-researcher5]

The area of pain care for people with profound intellectual disabilities was recognised as such a challenging and important task that the co-researchers expressed a need for increased support and expertise.

“I think it would be good if we had a specialist nurse who will deal with pain management alone, as you can under-diagnose, misdiagnose or over-diagnose pain” [Co-researcher 3]

### Medicating pain – oversimplified and undervalued?

The prescription and review of appropriate medication for the level of pain an individual may be in, was described as an unmet need.

“I would like to see appropriate medical interventions depending on the type and severity of the pain. So, I worked with somebody before, and he had a fractured jaw. He was prescribed paracetamol, so that is what he was getting” [Co-researcher 3]

### Dreamed practice

The co-researchers were asked to imagine what the very best practice might look like in this area of care, if there were no barriers or constraints. The following provides a summary of what was envisioned by the co-researchers:

• That pain care for this population would be recognised as a priority.
• That every individual with a profound intellectual disability would always have their pain and distress recognised and assessed, independent of who was working with them, or what relationship they had.
• That there would be an individualised, formal method of holistic pain recognition and assessment for this population.
• That there would be a method of formally and successfully sharing their unique knowledge of an individual within this population with other professionals.
• That there would be appropriate methods of assessment for this population in an emergency situation.

### Design

There were five focus group interviews and two individual interviews conducted in this phase of the study (see Table 2). After uncovering the best of current practice and shared visions of future best practice; the co-researchers reflected and designed interventions to work towards these visions.

This area of care was viewed as such a substantial challenge that initially the co-researchers were concerned about the achievement of dreamed practice being an impossible task in its totality. After much discussion around the aims of the study and of appreciative inquiry in improving care, rather than achieving the ideal, the co-researchers became very motivated and enthusiastic.

“We should be driven. We should be motivated in making this thing happen”

[Co-researcher 3]

The co-researchers came to the conclusion that the development of a holistic pain recognition and assessment tool, uniquely suited to people with profound intellectual disabilities, would assist in reducing the challenges to this area of care. The group also recognised that there was a need for the importance of pain care to be highlighted within the organisation, to create awareness of the multifaceted pain experience of people with profound intellectual disabilities. The co-researchers expressed this as a method of commencing dialogue with all staff in their respective care areas, increasing the likelihood of appropriate pain management for this population. Two co-researchers expressed an interest in taking a leading role in the development of a pain awareness campaign for people with profound intellectual disabilities in the organisation.

### Development of a pain awareness campaign

**Figure 2.**
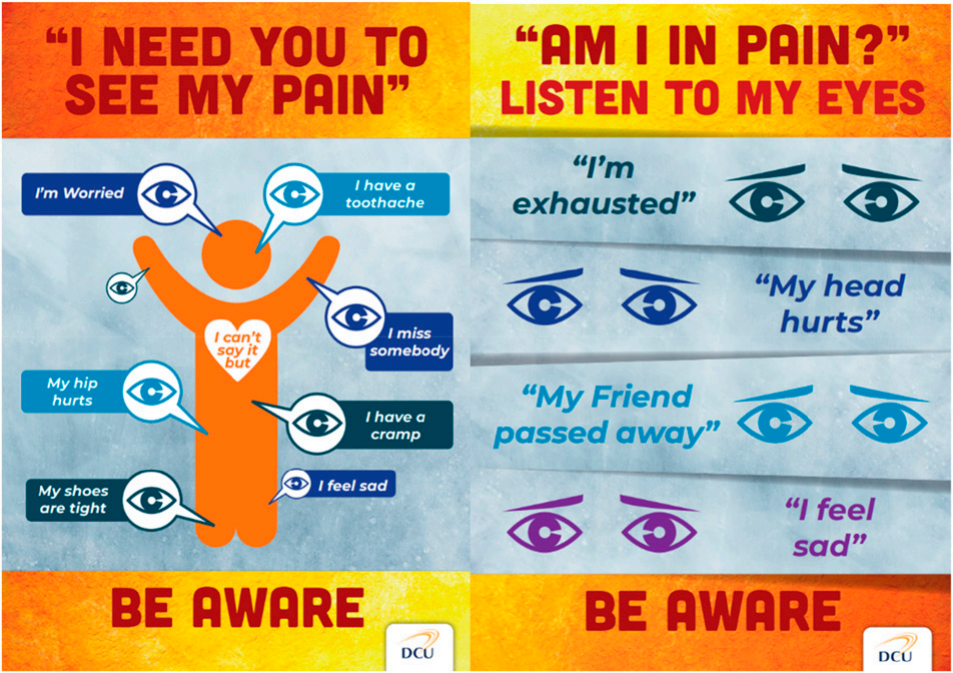
Awareness Posters.

**Figure 3.**
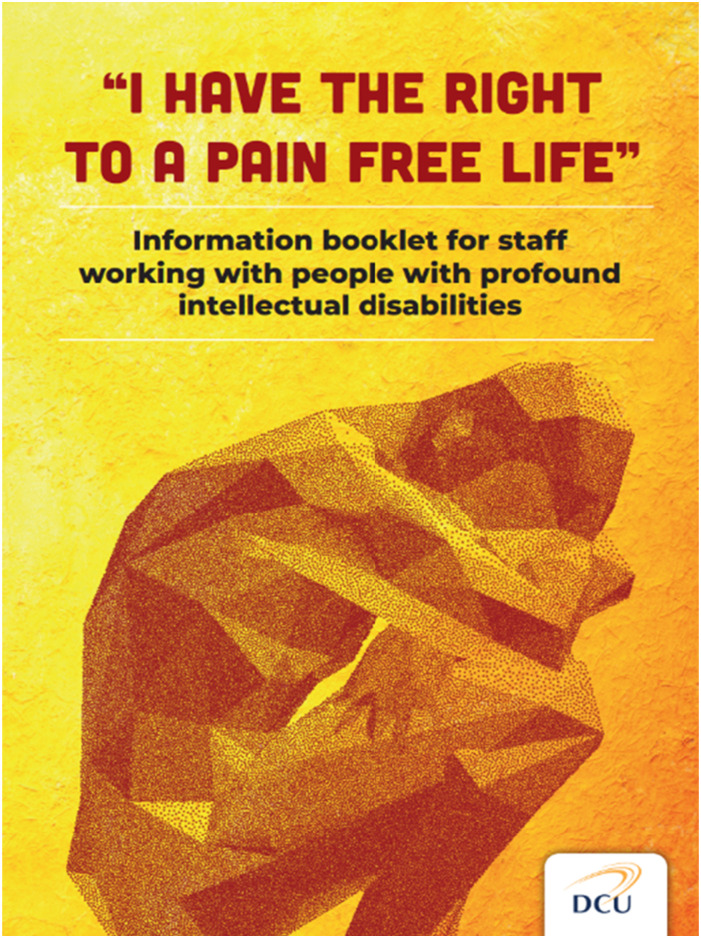
Awareness Booklet.

There were two focus group and two individual interviews conducted for the development of a holistic pain awareness campaign for individuals with profound intellectual disabilities in the organisation. There was also regular communication through email during the design of resources. Creative methods were adopted during the focus group interviews, where the co-researchers were asked to draw their ideas for pain awareness posters. They then shared and discussed these, choosing two poster ideas. MG had these developed by a graphic designer and the co-researchers reviewed these a number of times (see Figure 2). The co-researchers and MG also developed a pain awareness booklet titled; ‘I have the right to a pain free life’ information booklet for staff working with people with profound intellectual disabilities (see Figure 3). Through thorough discussion, a number of topics to be addressed in the booklet were decided upon. These included; the interconnection of different pain facets, common painful conditions, communication, pain threshold, assessment tools, pharmacological pain care, non-pharmacological interventions. The booklet was again designed by a graphic designer and reviewed by the co-researchers and MG.

### Development of a holistic pain recognition and assessment tool

There were four focus group interviews, specifically for the development of a holistic pain recognition and assessment tool for this cohort. The co-researchers discussed the need to first recognise that an individual in this cohort was in pain, for it to be assessed. There was a consensus between the co-researchers that pain is not being assessed and managed appropriately for individuals in this population, primarily due to a lack of recognition of the presence of pain. Initially the co-researchers had ideas that related to the specific individuals under their care, but through discussion from different viewpoints and differing areas of care, the co-researchers could focus their attention on developing a tool that may work universally for this population.

“It was good to see other people’s take on it as well, and they will relate it to their service users….. I’m coming from my head…. seeing somebody else with different experiences as well. It’s very good” [co-researcher 4]

The co-researchers recognised the importance of including all forms of pain and distress within the tool. They expressed that although some people with profound intellectual disabilities may communicate their pain using the same method, independent of the cause, that this in itself would be a necessary and helpful thing for staff to know. Through an understanding of the challenges to the use of a tool from the discovery phase of the study, the co-researchers with MG, decided upon the development of a one-page tool that is completed collaboratively by a team of people working with the individual. A co-researcher asked two staff members to complete the RAPPID tool (Recognition and assessment of pain in people with profound intellectual disabilities), during its development, separately for the same individual with profound intellectual disabilities under their care. The completed tools showed differences in the description of behavioural vocabularies and signs of pain in this individual, compounding the importance of collaborative completion of the tool; as different carers may recognise different signs of pain in the same individual. The guidance developed for the tool was to review it yearly or sooner if needed and for it to remain in an accessible place such as a handover file, easily highlighted to relief or new staff.

**Figure 4.**
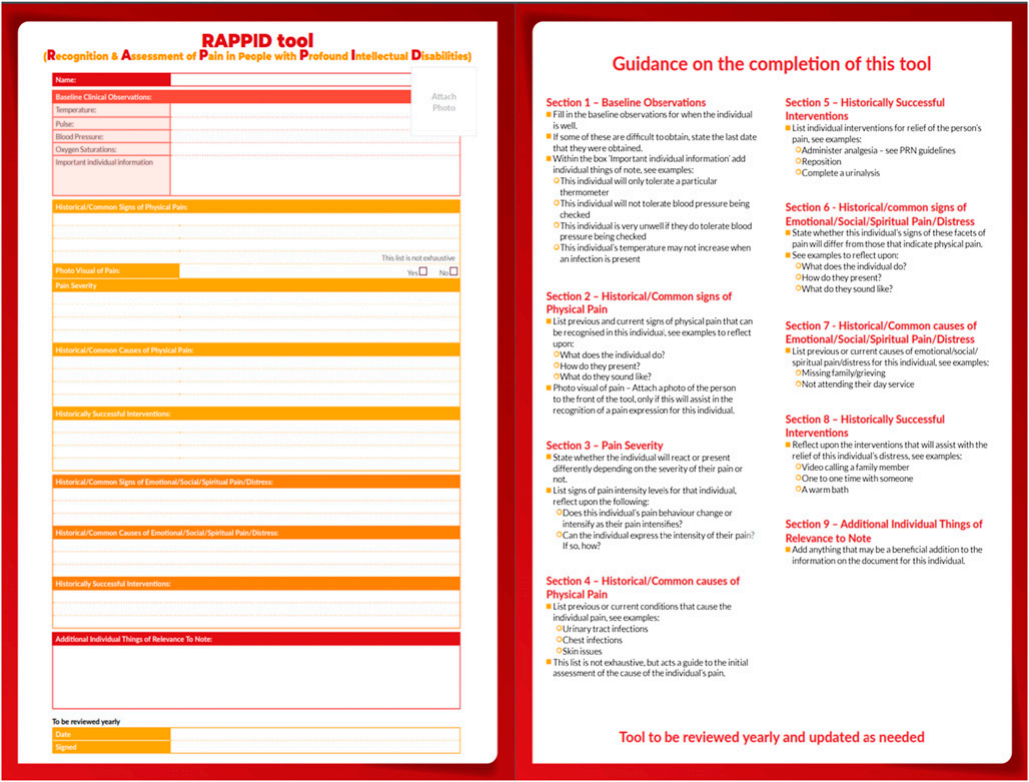
RAPPID tool.

During the tool development process, the co-researchers took drafts to their staff teams for review, bringing their feedback to the focus groups. Using this feedback, adaptations were made, including a guide to completing the tool on its back page. The tool was created as an individualised tool for use within different care areas and situations. It acts as an initial guide to recognising an individual's distress, followed by a guide to assessment and management. It can assist with emergency care, through the inclusion of individual medical and behavioural information. It was named the RAPPID tool (Recognition & Assessment of Pain in people with Profound Intellectual Disabilities). The tool was designed by a graphic designer (see Figure 4) and is coloured with bright, eye-catching colours.

The significance of this tool is that it is fully individualised and would be available for all staff on a daily basis. It is not to be completed when pain is suspected but acts as a guide to recognising an individual with profound intellectual disabilities communication of their pain or distress; enabling early intervention.

### Destiny

There were two focus group and three individual interviews for this phase of the study. The multifaceted pain awareness campaign posters and booklets were emailed to all managers, in all services of the organisation. It was requested within these emails that management display these and discuss them at staff meetings. The posters were also displayed in the central offices and clinics of the organisation. A pilot of the RAPPID tool is underway.

## Discussion

### Personhood in pain care

Nursing models of care have developed and transformed over time (Bolten and Gillette, 2019). A biopsychosocial model now guides intellectual disability care, incorporating holism and person centredness (Moulster et al., 2019). These are core values of intellectual disability nursing (McCarron et al., 2018), which must be integrated into all areas of care, including pain care. To overlook the multifaceted nature of pain, is to disregard an individual’s personhood. People with profound intellectual disabilities are whole people, with individuality, abilities and life experience, as is the case with people from the general population (Vaurhaus, 2014). Formal carers coming into contact with people with profound intellectual disabilities have a responsibility to respect these aspects of the person, in order to give best care. The resources produced in this study acknowledge and reflect the uniqueness of the pain experience of the person with profound intellectual disabilities. Without a respect for and recognition of each individual’s personhood and thus their complex responses to pain, they may remain in pain and distress. A close, trusting relationship between carers and individuals with profound intellectual disabilities is essential for best care as unfamiliar carers may not be able to interpret the person's mode of communication (Nieuwenhuijse et al., 2024). It is recommended by Nieuwenhuijse et al. (2024) that although self report is considered the gold standard of care, proxy reports by familiar carers of people with profound intellectual disabilities should be valued as best practice, as part of a collaborative decision making process where appropriate. However, this relies on the presence of these carers who have expert knowledge of the individual. Within this study, the concerns of unfamiliar staff not having the resources to assist with the recognition of pain in this cohort and the challenges to communicating familiar carer knowledge with others was seen as a barrier to best practice.

### Broadening perspectives on pain

The inclusion of all aspects of the interconnected, multifaceted pain experience in the awareness resources and RAPPID tool (Recognition and assessment of pain in people with profound intellectual disabilities) development allowed for staff to reflect on their current practices and encourage a holistic interpretation of pain care. There was an expression of respect for each individual with profound intellectual disability’s personhood within this study, through deep discussion on the individuality and abilities of individuals in this cohort to communicate, and experience all facets of pain and distress. However, the perceived risks associated with physical pain and fear of not recognising illness in a timely fashion, created a strong belief in the importance of prioritising the recognition and assessment of physical pain. Each co-researcher within this study had experience of people under their care developing preventable illness and even death. These past experiences of personal distress associated with missed care, increase feelings of powerlessness. It is evident that the early recognition of physical pain is imperative, however pain is not unidimensional. Emotional pain can be misinterpreted as physical pain in these individuals (Adams and Jahoda, 2019). A physical pain experience can also be affected or altered by an emotional, social or spiritual experience (Davis, 2000; Saunders, 1993; Sulmasy, 2002). A multimodal approach to the relief of pain is optimal (Bolten and Gillette, 2019). An awareness of the interconnection of all facets of pain will assist in the treatment or management of an individual’s pain and distress (Doody and Bailey, 2017).

In order for this population to be observed and assessed for pain and distress, there must firstly be a belief and awareness of the possible presence of the subjective, multifaceted experience. A spotlight was shone on the importance of ‘seeing’ suffering, through the development and distribution of the awareness campaign resources developed in this study. There are historical beliefs of people with intellectual disabilities not experiencing pain or having a higher pain threshold than those in the general population (Symons et al., 2008). Although the relief of suffering is a fundamental aspect of nursing care (Cassell, 2004), it was highlighted within this study that a belief of a reduced pain experience for people with profound intellectual disabilities is still present among some individuals today, through storytelling of the co-researchers experiences through their nursing careers. This pain belief may be compounded by the fact that individuals with profound intellectual disabilities may not react to pain and distress in an obvious or typical fashion (Courtemanche et al., 2012; Icht et al., 2021). Creating and distributing pain awareness resources aimed to challenge these historical beliefs. The international association for the study of pain (2020) has stated that pain may be present without verbal reports of such. A study by Bernaal-Celestino et al. (2022) showed that only 36.8% of individuals with communication difficulties receive pain relief, even after pain is detected.

There are multiple communication challenges in the area of pain care for individuals with profound intellectual disabilities (Chadwick et al., 2019). This study highlighted these challenges as issues with interpersonal communication between nurses and the individuals within this cohort, issues with the communication of knowledge between nurses within the direct care setting and issues with communication between different professionals. In many ways people with profound intellectual disabilities are dependent on carers (Bellamy et al., 2010), however the co-researchers within this study explained that all people within this cohort can communicate, through storytelling. Oberlander and Symons (2006) discuss behavioural communication methods as an alternative method of self-report. It is the role and responsibility of the nurse to learn to understand these methods and react appropriately (Vorhaus, 2014). Challenges to self-reporting internal states also affect the recognition of forms of distress such as depression (Eaton et al., 2021). These internal states can be misinterpreted as physical pain and be treated with analgesia, creating the possibility of ineffective care. As a close and trusting relationship is essential for good communication and understanding between the individual with profound intellectual disabilities and the nurse, new or irregular staff may miss pain care. This risk causes moral distress to nurses who know these individuals, and feel powerless to create change (Cho et al., 2020). Addressing these communication barriers through the empowering process of the development of the resources may have multiple benefits, including significantly improved pain management, reduced staff distress, increased nursing job satisfaction and retention. This will be an area for future inquiry.

Behaviours that challenge in individuals with intellectual disabilities have historically been managed with antipsychotic medications, without any diagnosis of psychiatric conditions (Deb et al., 2023). These medications can cause many adverse events and there is a growing awareness of the need for these medications to be discontinued for large groups of individuals within services (de Kuijper and Hoekstra, 2018). Behaviours that challenge are often used by people with profound intellectual disabilities as a mode of communication, for example hand biting, head hitting, screaming. When carers find alternative methods to assist the individual in communicating their needs, these behaviours have been seen to reduce (Muharib et al., 2021). The RAPPID tool will aim to assist nurses and staff to recognise each individual with profound intellectual disability’s communication of their pain, reducing the individual’s frustration and improving pain care. The pain awareness campaign aims to assist staff to assess all aspects of the person, moving towards the recognition of the source of their distress, which should direct staff to appropriate ways of managing the specific issue at hand.

### Communicating pain care

Within the study, the co-researchers discussed their knowledge of different individuals with subtle and atypical communication methods. There was an emphasis on the importance of relationship and time spent with the person, to understand and adapt to their methods of communication. This has not been prioritised traditionally in this area of care due to the challenges of sharing unique knowledge of individuals between staff. The RAPPID tool was developed to address this challenge, as it allows for subtle and individual communication styles of people within this cohort to be communicated more effectively. The current tools available for use with people who are non-verbal, list behavioural expressions of pain that are generalised and more easily interpreted than the often atypical and individual methods of pain expression in each person with a profound intellectual disability (Lotan et al., 2010; Solodiuk et al., 2010; Van der Putten and Vlaskamp, 2011). The DISDAT (Disability Distress Assessment Tool) was developed for people with more severe communication issues (Regnard et al., 2007), allowing for a distress assessment by the comparison of an individual’s presentation while distressed or content. The RAPPID tool differs from this as it has a focus on the initial recognition of pain for the person by any carer, with additional guidance on further assessment and pain management. The tool is fully individualised and holistic, reducing the risk of reductionist pain assessment for individuals with profound intellectual disabilities causing them to experience a loss of their personhood and remaining in unnecessary pain or developing serious illness. The RAPPID tool includes the previous reasons for an individual's distress and methods used to manage or relieve these, which is not available in other current tools. This takes into consideration people with profound intellectual disabilities’ personhood. It was discovered through the study that there was a need for a practical and efficient tool, completed collaboratively for daily use, and for use in emergency situations. The RAPPID tool meets this need. It was developed as a one page tool, completed collaboratively by a team of carers which can be made available for each staff member daily. It can be used in emergency situations, as specific individual information for each person within this cohort that will assist with medical assessments is included.

The co-researchers discussed their own abilities to recognise pain and distress in the individuals under their care as tacit knowledge. This aesthetic pattern of knowing in nursing (Carper, 1978) has remained as the primary method of recognising, assessing and reviewing pain for this population. This is due in part to the challenge of formalising methods of communicating informal and expert knowledge between professionals (Goodall et al., 2023). If tacit knowledge is not afforded the validity that logical knowledge is, and formal methods of recognising distress in individuals with profound intellectual disabilities are not available, there are increased risks of pain not being managed for people within this population. The RAPPID tool provides a formal method of information sharing, and validating tacit knowledge about the individual and their pain. It attempts to give credence to the often subtle methods of recognising distress in individuals within this cohort, which is imperative for a feeling of autonomy and empowerment for nurses.

### A journey to empowerment

Appreciative inquiry, as a research philosophy, aims to empower the co-researchers to create positive change, and this has been evident in previous nursing research (Surani et al., 2019). Empowering nurses through bestowing a sense of autonomy, control and confidence in their own competence will encourage improvements in practice (Qtait, 2023). Using this approach to research within this study, the co-researcher’s sense of empowerment and control grew and evolved over the four phases as they led the changes in practice.

There was a fifteen percent reduction in intellectual disability nursing staff in Ireland between 2016 and 2019, and this is a continuing trend (Doyle et al., 2023). Individuals with intellectual disabilities are a stigmatised population (Pelleboer-Gunnink et al., 2021; Scior et al., 2020). Mitchell (2000) explores the concept of “parallel stigma” (p. 78) between intellectual disability nurses and the intellectual disability population. This may be due in part to a lack of regard for or value placed by society on individuals with intellectual disabilities, which also reflects on those who care for them (Pearce, 2023). There can be a lack of understanding of the unique qualities and relational skills of the intellectual disability nurse by other professionals. The nurses caring for individuals within this population can have a negative view of their own worth and ability to create change, in response to this (Doyle et al., 2023). There was a reflection of this within this study, as there were initial expressions of overwhelming powerlessness in the communication of concerns relating to individuals with profound intellectual disabilities to other professionals. This can create burnout and cause staff retention problems (Doyle et al., 2023). Nurses who feel disempowered in their work will experience lower job satisfaction (Li et al., 2018). This disempowerment can be caused by missed care (Li et al., 2018) due to factors that are felt to be out of the nurse's control (Janatolmakan and Khatony, 2022). Pain care has been highlighted within this study as a recognised area of missed care for people with profound intellectual disabilities. The care of individuals with profound intellectual disabilities is a challenge for a multitude of reasons but effective care is possible, and nurses working directly with this population can lead this.

The co-researchers in this study engaged in collaborative discussion on the skills of nurses and the power that they held as pioneers for change in pain care for individuals with profound intellectual disabilities. There was a shift from overwhelm to positivity, which allowed for the creation of change. The nurses within this study are now moving forward with a sense of hope and positivityfor best pain care practice. The RAPPID tool can allow for the innate and aesthetic knowledge nurses have of individuals with profound intellectual disabilities to be formalised. This will allow for improved communication with carers and professionals who may not have a relationship with or deep knowledge of an individual. The co-researchers within this study expressed that this more formal method of pain recognition and assessment may empower nurses in the field of intellectual disability care to have their skills and abilities validated; ultimately improving care outcomes for individuals with profound intellectual disabilities.

### Limitations

The results need further validation and the tool needs to be used and tested in another study such as an outcomes based observational study and in further settings to see if the benefits seen by these staff continue over time and in different organisations with different challenges and organisational structures.

## Conclusion

There is an evident need for improvements in pain recognition and assessment for this population. A holistic approach to the relief of pain and suffering, with a respect for each individual with profound intellectual disabilities’ personhood is essential. People with profound intellectual disabilities must be viewed as whole people who experience the full and interconnected range of pain facets, in order for these individuals to receive best care. This study has aimed to create a greater awareness of this amongst staff who care for individuals within this cohort, and to develop a tool that encompasses the recognition, assessment and management of the multifaceted experiences of pain and distress. The RAPPID tool is an accessible, collaborative method of formally sharing unique knowledge of individuals with profound intellectual disabilities. This will require further formal testing. This study has produced a tool which is deeply embedded in the practice of experienced nurses, valuing and formalising their tacit knowledge about pain behaviours in this complex area of care. Appreciative inquiry, as a research methodology has been shown in this study to be effective in the empowerment of nurses, creating positive commitment to change.
